# The Rich Get Richer: Brain Injury Elicits Hyperconnectivity in Core Subnetworks

**DOI:** 10.1371/journal.pone.0104021

**Published:** 2014-08-14

**Authors:** Frank G. Hillary, Sarah M. Rajtmajer, Cristina A. Roman, John D. Medaglia, Julia E. Slocomb-Dluzen, Vincent D. Calhoun, David C. Good, Glenn R. Wylie

**Affiliations:** 1 The Pennsylvania State University, Department of Psychology, University Park, Pennsylvania, United States of America; 2 The Pennsylvania State University, Department of Mathematics, University Park, Pennsylvania, United States of America; 3 Hershey Medical Center, Department of Neurology, Hershey, Pennsylvania, United States of America; 4 The Mind Research Network, Albuquerque, New Mexico, United States of America; 5 Kessler Foundation Research Center, West Orange, New Jersey, United States of America; University Of Cambridge, United Kingdom

## Abstract

There remains much unknown about how large-scale neural networks accommodate neurological disruption, such as moderate and severe traumatic brain injury (TBI). A primary goal in this study was to examine the alterations in network topology occurring during the first year of recovery following TBI. To do so we examined 21 individuals with moderate and severe TBI at 3 and 6 months after resolution of posttraumatic amnesia and 15 age- and education-matched healthy adults using functional MRI and graph theoretical analyses. There were two central hypotheses in this study: 1) physical disruption results in increased functional connectivity, or hyperconnectivity, and 2) hyperconnectivity occurs in regions typically observed to be the most highly connected cortical hubs, or the “rich club”. The current findings generally support the hyperconnectivity hypothesis showing that during the first year of recovery after TBI, neural networks show increased connectivity, and this change is disproportionately represented in brain regions belonging to the brain's core subnetworks. The selective increases in connectivity observed here are consistent with the preferential attachment model underlying scale-free network development. This study is the largest of its kind and provides the unique opportunity to examine how neural systems adapt to significant neurological disruption during the first year after injury.

## Introduction

Over the past decade, functional brain imaging has dramatically changed the scope of investigation in the study of neurological disorders like traumatic brain injury (TBI). Even with considerable attention given to methods such as functional MRI and the study of cognitive, motor and sensory deficits in TBI, there remains much unknown about recovery of function after TBI in particular from a systems neuroscience perspective. Recent developments in network connectivity have broadened the scope of investigation, providing unparalleled opportunity to examine whole-brain communication after significant neurological disruption. One applied mathematical approach, graph theory, has received significant recent attention in literatures using functional brain imaging methods (e.g., functional MRI) to examine the flow of information in dynamic networks. While graph theory has a much longer history in the areas of chemistry [1]–[2] and in the early 1900s in the social networks [3], in its relatively brief application to the neurosciences, this approach has already influenced how we conceptualize network communication. In particular, graph theory analyses in animals [4] and functional imaging studies in humans [5]–[7] demonstrate that neural systems hold “small-world” properties characterized by high *clustering* or the presence of densely linked sub modules in the graph, while also retaining short net communication paths between pairs of nodes. The small-world structure affords specialized processing of information locally while simultaneously permitting large-scale information transfer throughout the network [8]. It is a goal in the current study to examine network changes occurring after moderate and severe traumatic brain injury (TBI) through graph theory analysis. Examining whole-brain connectivity dynamics will provide previously unavailable information about how neural systems adapt to catastrophic disruption.

### Clinical network neuroscience

In the clinical neurosciences it remains an important goal to understand the basic brain changes associated with neurological disruption and the implications these changes have for behavioral deficit and recovery trajectory. There has been widespread use of functional imaging methods to examine task-related brain changes (e.g., mean signal differences) in localized regions of the brain but there has been a recent shift to explore the covariance (i.e., connectivity) between brain regions in addition to fundamental signal amplitude changes.

With the more recent emphasis in connectivity modeling in functional neuroimaging, there is an expanding literature documenting the network alterations associated with brain injury and degenerative processes (see 9). Several studies to date have demonstrated that neurological disruption results in altered connectivity in large-scale neural networks [10]–[15] including evidence that even focal injury has widespread consequences for broader network functioning [16]–[17]. For example, both focal and diffuse injuries observed in TBI may disrupt distal connectivity which is a distinct and crucial feature to the small-world topology required for efficient transmission of information in neural systems [12]–[13]. While it would appear a paradoxical consequence to physical network disruption, we have observed that a primary response to neurological disruption in dynamic systems is hyperconnectivity [12], [18]–[19]. In this paper we aim not only to determine if hyperconnectivity is observable during the first 6 months post injury, but to also determine the specific sites (if they exist) where hyperconnectivity is likely to be observed.

In determining which networks may account for hyperconnectivity after injury, work outside the neurosciences has demonstrated that the small-world topology is particularly resilient to non-selective or “random” connectivity loss [20]–[21]. These authors also demonstrate however that that targeted “attack” on critical network hubs can lead to catastrophic consequences for network communication. Hubs provide a buffer to network disruption and similar effects may also be expressed in biological systems. For example, the focused loss of anterior-posterior connectivity (e.g., frontal to PCC to hippocampal connections) in Alzheimer's has devastating consequences for functioning in the areas of memory, spatial navigation, and maintaining semantic associations [9]. By comparison, the pathophysiology occurring in TBI is selective for certain regions (e.g., temporal and frontal poles), but does not function as a targeted attack on connections between essential subnetworks (e.g., default mode network, DMN) thus permitting the opportunity for their greater integration. We hypothesize that hyperconnectivity induced by injury will be expressed in the brain's most highly connected regions, or the “rich club”, a high capacity but metabolically expensive network that forms the backbone for efficient information transfer in the brain's various subnetworks [22]–[23]. In order to examine the influence of TBI on network hubs, we will make use of functional MRI and graph theory to examine whole-brain connectivity in TBI early after injury. In doing so, this will be the first study to examine the effects of TBI on neural network hubs over the course of early recovery in moderate and severe TBI.

### Network Analysis

Possibly the most important early decision in network modeling is determining the nodes, or brain regions, that will contribute to the model. In large-scale network analyses, the characterization of the network nodes has a direct influence on the graph properties observed [8], [24]. Recent efforts to examine “small-world” properties in TBI have used 20–30 ROIs to create unweighted (i.e., binary) networks [14], [17], [25]. Anatomical ROIs are often used to avoid biased selection and circularity in data interpretation [26]; yet these approaches aggregate a number of functionally distinct signals within each ROI. For example, Brodmann's area 46 is one of the largest ROIs in anatomical atlases and maintains critical roles in a number of functions, yet in the absence of additional parcellation, the hundreds of voxels that can be sampled this region are averaged and treated as a single homogenous signal. To address these concerns, we use a data-driven approach for ROI parcellation through the use of spatial independent component analysis [27]–[28]. Each ROI is represented as a functional signature as opposed to an anatomically bound average of many functional signals [29]. We anticipated that the approach used here will be sensitive to the network changes associated the early recovery window in TBI. Moreover, in studies using fMRI to examine neurotrauma there is concern regarding the influence of brain lesions on the BOLD signal [30] and this is particularly problematic in local areas of hemorrhage where blood products cause susceptibility artifact and local signal attenuation [31]–[32]. However, the ICA procedure implemented here can isolate the effects of local signal drop-out as a “component” and model these data or remove the signal during “denoising and nuisance” identification. This approach addresses basic differences in brain morphology and local signal drop-out due to the effects of TBI early after injury.

### Study Goals and Hypotheses

There are two hypotheses in this study. First, we propose that a common response to moderate and severe TBI during the first few months post injury is *hyperconnectivity*, or increases in the magnitude and/or number of connections. We test this hypothesis by examining both the number and strength of connections in the TBI sample over time as compared to a health control (HC) sample. Second, we hypothesize that enhanced connectivity during recovery will occur in three of the most highly connected subnetworks, or “rich club”: the salience network (SN, e.g., anterior insula), the executive control network (ECN, e.g., dorsolateral prefrontal cortex and parietal cortex) and DMN (e.g., PCC and medial frontal cortex). There are three sources of evidence for this. First, in the work examining fMRI signal amplitude change during task, the most common finding is increased involvement of the ECN, or the PFC and parietal regions after TBI [33]–[35]. Second, there is recent evidence that the PCC and its distinct roles within the DMN has critical function in integrating other subnetworks and facilitating information transfer across a broad spectrum of neurological disorders [36]. Finally, recent work has demonstrated that TBI results in increased connectivity to the insula which maintains a central role in the salience network [37]–[38]. We tested this second hypothesis by examining the nodes most likely to show enhanced connectivity during recovery from TBI. Finally, given the relationship between the DMN and SN and cognitive performance [39], we also anticipated that hyperconnectivity in these networks would predict performance deficits on tests of processing speed and working memory, two critical areas of cognitive dysfunction after TBI [40]–[41].

## Method

### Subjects

Study recruitment included 22 individuals with moderate and severe TBI between the ages of 18 and 53 years and 15 healthy adults of comparable age and education (see Tables 1 and 2 for demographic and clinical information). Due to significant frame-by-frame head motion identified via ArtRepair [42] one individual with TBI was removed from the study, leaving a total study sample of 36 individuals at two time points. All study participants underwent two MRI scanning sessions separated by approximately three months. For the TBI sample, initial data collection occurred at three months after emerging from posttraumatic amnesia (PTA), or a period of confusion and amnesia following coma emergence, and the second scanning session followed three months later. These 3- and 6-month windows for measurement are consistent with animal studies examining “very long” outcome [43]–[48] and the TBI “outcome” literature based upon timepoints where significant change is expected behaviorally [49]–[55]. TBI severity was defined using the Glasgow Coma Scale (GCS) in the first 24 hours after injury [87] and GCS scores from 3–8 were considered “severe” and scores from 9–12 were considered moderate. In three cases, participants were included with a GCS score of 13–14 because acute neuroimaging findings were positive. Participants were excluded if they remain in treatment for concomitant spinal cord injuries, orthopedic injury, or other injury making it difficult to remain still in the MRI environment. So that findings were generalizable to a typical moderate and severe TBI sample, patients with focal contusions and hemorrhagic injuries were included unless injuries required neurosurgical intervention and removal of tissue resulting in gross derangement of neuroanatomy. Research was conducted with approval by institutional review board and Office of Human Subject Protection at the Pennsylvania State University (PSU). Informed written consent for all participants was obtained at the time of study enrollment. The current study includes individuals who may be cognitively impaired, so capacity for enrollment in the study was based upon how decisions were being made for medical treatment and for functioning independently. If an individual retained capacity to sign for medical procedures and functioned independently (i.e., lived alone, retained driver's license), consent to participate was accepted; however, if caregiver signature was required for medical procedures or the potential participant was not functionally independent this signature and a signature of assent by the potential participant were similarly required for study enrollment. PSU is positively and unequivocally committed to the promotion, encouragement, and facilitation of academic and clinical research in the broad area of general or specific measurements of human development, health, and performance. PSU is dedicated to the ethical treatment of human participants in all research activities conducted under the auspices of this institution and assumes responsibility for safeguarding their rights and welfare.

**Table 1:** Demographic descriptors, injury information.

Demographic	Traumatic Brain Injury Mean (sd), n = 21;	Healthy Controls Mean (sd), n = 15
**Age (years)**	27.9 (9.1)	28.8 (11.9)
**Education (years)**	12.5 (1.6)	13.4 (1.7)
**Gender**	18 M, 3 F	9 M, 6 F
**Race/Ethnicity**	Caucasian, n = 14; African American n = 4; Hispanic, n = 2; Asian, n = 1	Caucasian, n = 11; African American n = 3; Hispanic = 1; Asian = 0
**Glasgow Coma Scale** 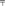	mean: 7.1, min: 3; max: 14, mode: 3	-
**Time-post injury (days)**	113.5 (32.3)	-
**Time between scans (days)**	106.2 (24.6)	116.2 (33.9)

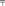
GCS when available 15/22 cases; when not available, inclusion based upon positive CT finding. No between-group differences observed for age, education, gender, ethnicity, or Time between scans.

**Table 2:** Performance on cognitive testing.

Behavioral testing	TBI Time 1 mean (sd), n = 21	TBI Time 2 mean (sd), n = 21	Healthy Controls mean (sd), n = 15
**Digit span - forward**	10.6 (2.4)	10.7 (2.0)	10.8 (1.7)
**Digit span - backward**	6.19 (1.4)	7.0 (1.7)**	6.07 (2.1)
**Digit span - total**	16.8 (3.2)	17.7 (3.3)	17.5 (2.9)
**VSAT – letter**	55.4 (15.6)	62.3 (16.7)**	71.5 (17.7)^‡‡^
**VSAT - symbol**	52.2 (14.4)	60.8 (14.9)**	73.2 (20.7)^‡‡^
**VSAT - total**	107.7 (29.0)	123.2 (29.5)**	144.8 (37.4)^‡‡^
**Trails A**	26.7 (11.5)	27.8 (10.3)	20.0 (11.1)
**Trails B**	89.2 (57.2)	66.9 (35.8)**	60.5 (28.1)^‡‡^
**Color-word: Color**	48.8 (21.4)	46.9 (23.7)	38.4 (18.0)^‡^
**Color-word: inhibition**	83.1 (25.2)	78.4 (26.7)*	78.7 (31.1)

Between-time differences significant at *p<.05, **p<.10, between-group differences significant at ^‡‡^p<.05, ^‡^ p<.10. Note: Between group comparisons made for Time 1 only.

### Cognitive Assessment

The most common cognitive deficits following TBI are in the areas of working memory and processing speed [56]–[58]. All participants completed a brief battery of tests assessing these areas of functioning to determine: 1) areas of cognitive deficit compared to a HC sample and 2) relationship between connectivity changes and cognitive deficit. To assess working memory and processing speed we used the visual search and attention task [VSAT; 59], the Stroop task [60]–[61], the Trail Making Test (A&B) [62]–[63] and the digit span subtest from the Wechsler Adult Intelligence Scale –Fourth Edition (Digit Span) [64]. Testing was completed at each data acquisition interval for the TBI sample and at Time 1 for the HC sample. Repeat testing has inherent problems with respect to the effects of practice and while prior exposure to the stimuli may have some small influence on Time 2 scores, the tests presented were chosen specifically because they show little practice effects (e.g., test-retest of the VSAT in healthy adults with a 2-month delay is r = 0.95; [59]). Moreover, tests of rapid decision making and information processing have been shown to demonstrate negligible practice effects when repeated after several months [65]. One method for controlling for practice effects is to compare to a HC sample also tested twice. However, comparisons with an HC sample to determine practice assumes equivalent learning/task acquisition between samples, yet there is a long history of research documenting slowed learning and task acquisition after TBI [40]. Therefore, it was not a goal to measure cognitive change in the HC sample over time, with the exception of the behavioral data collected during each of the fMRI tasks (i.e., 1-back) to verify stable cognitive status between time points.

### Focal lesions

There are often whole-brain structural brain changes even in cases of TBI where the primary injury is isolated (e.g., subdural hematoma), [66]–[67] and diffuse axonal injury (DAI) is a nearly universal finding [68]. Moreover, focal injuries can have widespread consequences for brain function; so focal injury was not an exclusionary criteria in the current study, unless the injury was so severe so as to require neurosurgical intervention (i.e., craniotomy) and/or gross derangement of neuroanatomy. Inclusion of cases where identifiable injury was evident permitted direct examination of TBI as it naturally occurs even in brain regions directly influenced by injury.

### MRI procedure and Data acquisition

Data were acquired using a Philips Achieva 3T system (Philips Medical Systems, The Netherlands, n = 8) with a 6-channel head coil, a Siemens Magnetom Trio 3T system (Siemens Medical Solutions, Germany, n = 13) with an 8-channel head coil both housed in the Department of Radiology, Hershey Medical Center, Hershey, PA, or a Siemens Allegra 3T MRI in the Department of Radiology at UMDNJ-NJMS in Newark, NJ, n = 15). Healthy and TBI samples were distributed between the MRI scanners and all subject data were collected on the same scanner over time to maximize intra-subject reliability.

Subjects were made aware of the importance of minimizing head movement during MRI scanning and trials containing significant motion were discontinued or repeated. High resolution brain anatomical images with isotropic spatial resolution of 1.2 mm×1.2 mm×1.2 mm were acquired using an MPRAGE sequence: 468.45 ms/16.1 ms/18°, repetition time (TR)/echo time (TE)/flip angle (FA), 250×200 mm^2^ field of view (FOV), and a 256×180 acquisition matrix. Echo planar imaging (EPI) was used for functional imaging and parameters were adapted for equivalence. Imaging parameters for EPI were 2000 ms/30 ms/89°, TR/TE/FA and a 230×230 mm^2^ FOV, 128×128 acquisition matrix. Efforts were made to maintain consistency in parameters between MRI scanning sites (e.g., TR was 2000) and investigators consulted one another during data collection to monitor for any changes in data acquisition. We made use of a single run of a working memory task, the n-back [69]. In order to maximize accuracy, prior to entering the MRI environment, each subject was exposed to the task and permitted a practice trial to promote accurate and efficient performance. Each run was 135 or 142 volumes of eight “on” blocks of the 1-back, a low load task requiring the subject to maintain consecutive matching stimuli in mind when presented a string of letters [69]. Greater detail regarding the task and data collection are consistent with previously published work [70].

### Data processing and region parcellation

**Figure 1:**
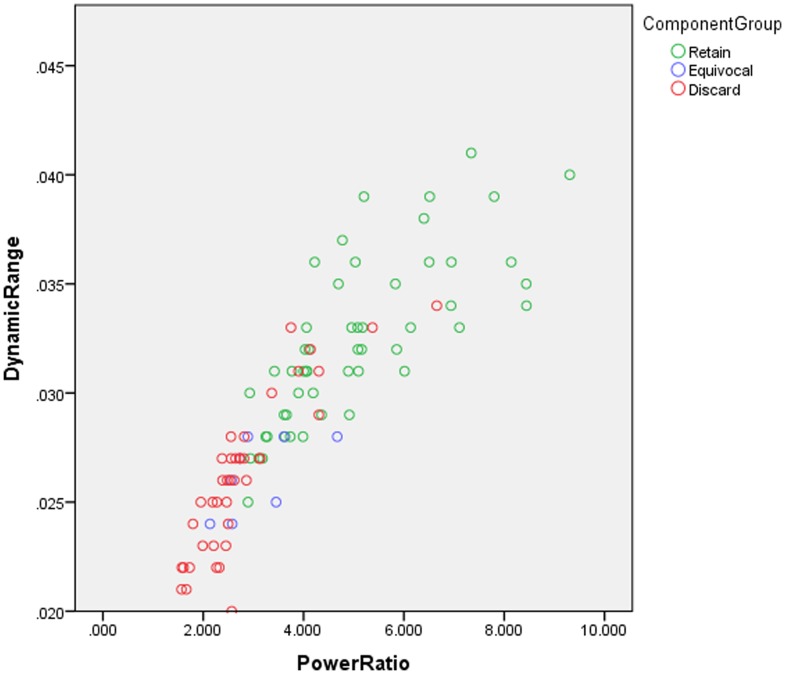
Data processing stream for fMRI pre-processing, ICA, and graph theory.

Figure 1 presents the processing stream for fMRI time series analysis. Initial steps of the processing stream involved pre-processing including slice-timing correction, realignment of the functional time series to gather movement parameters for correction, coregistration of the EPI data with a high resolution T1 image, and spatial normalization and smoothing [18], [70]. ArtRepair was used to identify slice and volume movement effects using the recommended cut-offs as a heuristic (5% slices and 25% volumes) [42]. Based upon these criteria 1 TBI subject showed significant frame-to-frame movement at Time 1, and was removed from the study.

### Independent component analysis

**Figure 2:**
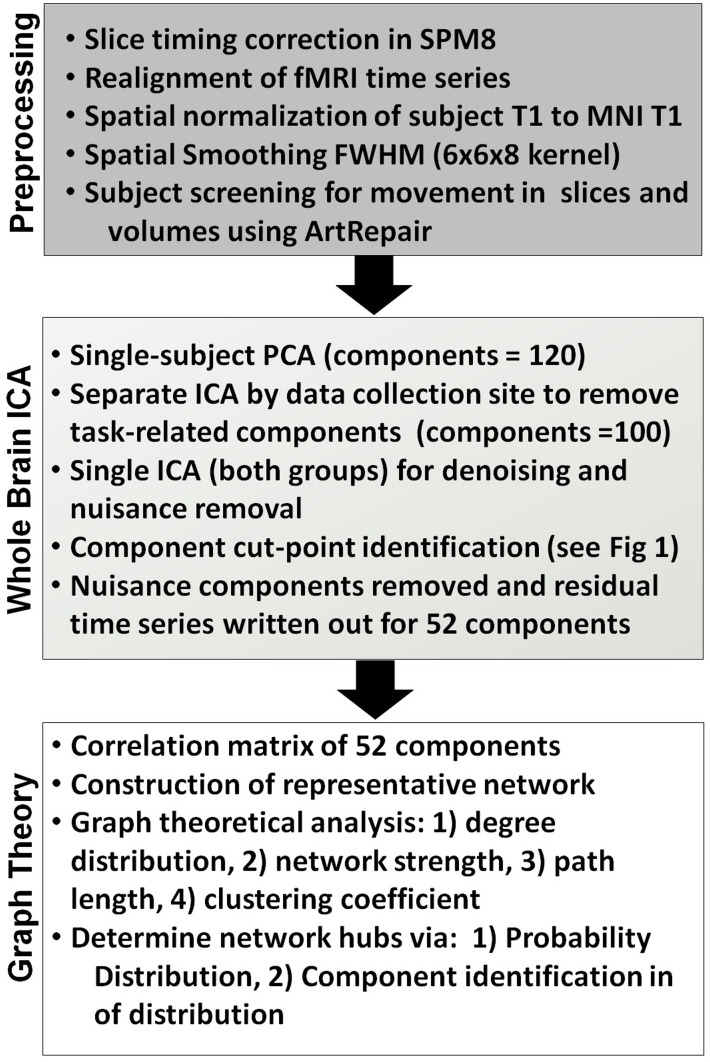
Component separation based upon dynamic range and low to high frequency power ratio. Dynamic range and power ratio for 100 components during inclusive ICA (all subjects). Rejected components (red) were determined by inspection of low to high frequency ratio and spatial extent consistent with Allen et al., (2011). In the primary analysis, “equivocal” components were discarded and the time series for the 52 remaining components composed the final graph. Supplementary materials include an additional analysis that included “equivocal” components in order to determine their influence on the graph and the results are nearly identical (see Table S1).

Group independent component analyses (ICA) were conducted using the Group ICA of fMRI Toolbox (GIFT). To achieve a detailed component structure and because a higher ordered dataset was desirable for graph theoretical analyses, we chose a relatively high model order ICA (100 components) for all analyses [71]–[75]. Subject-specific data reduction principal components analysis retained 120 principal components and group data retained 100 principal components. First, two separate ICAs were conducted to model two separate task timings for the hrf-convolved timecourses related to the influence of n-back performance. It was a goal to reduce the influence of task without removing relevant variance in the time series, so these initial ICA removed only the components with the highest regression coefficients (6–8 components) related to task. Then a second ICA was conducted including all subjects' residual timeseries in one group to provide the basis for the back-reconstruction to the individual level. Group-level ICA was chosen at this step because it has been demonstrated to be sensitive to individual effects while providing a framework for comparing the component structure across subjects [76]–[79]. Visual inspection of components was conducted by two raters and spurious components were removed using recommended guidelines for ICA [72], [80]. A heuristic cut point was set at a dynamic range of 2.5 and low frequency to high frequency power ratio of 3.0 [72]. To examine the consistency of this component rating, we conducted an inter-rater reliability check and agreement was very high for categorizing components as “retain”, “equivocal”, and “discard” (r>0.95). Figure 2 illustrates the result of component selection based upon the frequency ratio and dynamic range and the range of values for the 52 retained components. In addition, to guarantee that component selection did not influence the results of graph theoretical analysis, we also conducted an analysis that included 8 “equivocal” components, resulting in an additional graph of 60 components (referred to as FDR-60, see Supplementary Table S1).

Finally, we used a spatially constrained ICA (scICA) which provides a hybrid approach enabling us to focus on specific subnetworks of interest in this paper (i.e., the rich club) by providing a set of masks or images to the algorithm while also allowing the data to refine the resulting component [81]. The sICA approach in GIFT estimates maximally independent spatial sources from fMRI signal (see [72]) and maps the spatial extent and labels each component without the need for user identification (e.g., anterior insula- anterior salience network). Overall, we anticipate that the approaches used here provide safeguards for conservative data analysis and interpretation while retaining optimal sensitivity to dynamic network effects over time in TBI.

### Graph theory analysis

A representative network graph was created from the data parcellation described above, such that each *node* in the graph represented a resultant component of the whole-brain ICA [77]–[82], which is an approach previously used for connectivity modeling in clinical samples (see [83]–[84]). Pairwise correlations amongst all component time series were determined and, after thresholding using false discovery rate (FDR) at p<0.05 components with statistically significant correlations were joined by a weighted *link* in the network, where weights were determined as the value of the corresponding correlations [85]. Thresholding is a critical issue in creating a graph and has been shown to influence connectivity [86]. To address this issue a second graph was also created setting the lower bound threshold as the mean of the FDR corrected connectivity value from the HC Time 1 data during the first analysis (Sparse Graph, threshold: r = 0.403). This second, sparse graph provided the opportunity to examine connectivity in a graph composed of only moderate to highly connected nodes. The results of this graph analysis were largely consistent with the initial analysis (see Supplementary Table S2).

The original 52-component FDR-corrected graph was used in two primary sets of analyses. First, we tested Hypothesis 1 using whole-brain analyses of global graph properties. Graph metrics of interest included: a) the degree distribution, that is, the probability distribution of the number of links per node, b) total number and sum total weight, or strength, of network links, c) weighted clustering coefficient, and d) average global path length. Second, we tested Hypothesis 2 by a) examining change in node degree over time and b) identifying network hubs at each time point. Network hubs were determined as nodes of highest degree, calculated for a weighted network by summing the weights on all links incident to given node. Based upon 1 and 2 standard deviation thresholds, we examined these most highly connected regions at the individual level in order to determine: 1) whether the most highly connected nodes, or the tail of the degree distribution, were disproportionately represented in the TBI samples and 2) which nodes, or components, most commonly appear as hubs. Further, we examined the mean degree values for the most highly connected regions for each of the samples at each time point.

### Structural MRI analysis

In order to examine the morphometric changes associated with the TBI sample at time 1 and time 2, voxel-based morphometry (VBM) analysis was conducted using the VBM8 toolbox (http://dbm.neuro.uni-jena.de/vbm/). The initial processing step in VBM was used to quantify the white, gray and CSF compartments for all subjects via segmentation. During this processing stream, in order to maintain sensitivity to volumetric changes, we used non-normalized original high resolution T1 images for each subject and the TPM.nii tissue probability map (which is a modification of the ICBM Tissue Probabilistic Atlas) using a bias regularization of 0.001 (very light) and full-width-half-maximum 60 mm cutoff.

## Results

### Demographic and Neuropsychological Data

Tables 1 and 2 provide the demographic information and neuropsychological information for the two samples. The samples are comparable for age and education and the gender differences between samples was non-significant. There are two important results in Table 2. First, this TBI sample shows classic deficits in working memory and processing speed compared to the HC sample both at Time 1 and Time 2. Second, the TBI sample shows significant improvements on tests of working memory and processing speed between measurements. With respect to the 1-back task performed in the scanner there was little change in scores between time points, which we anticipate is due to the ceiling effects for RTs and elevated accuracy at the lowest n-back loads (TBI RT Time 1 mean = 698, sd = 115.8; TBI RT Time 2: 719.3, sd = 150.3; TBI Accuracy Time 1 mean = 88.5%, sd = 0.15; TBI Accuracy Time 2 mean = 87.7%, sd = 0.14). The HC sample also demonstrated similar performances over time (HC RT Time 1 mean = 648, sd = 122.6; HC RT Time 2: 644, sd = 161.6; HC Accuracy Time 1 mean = 92.8%, sd = 0.09; HC Accuracy Time 2 mean = 94.9%, sd = 0.07).

### MRI Volumetrics

Table 3 shows the group and time-point white matter, gray matter, and cerebrospinal fluid values that are the result of segmentation within the VBM suite (SPM8). The results predictably revealed little volumetric change between group and between time points. We anticipate that the 6-month window of time in this study is early to observe volumetric changes that are common to samples of chronic TBI [97]. The general consistency in brain volume between time points also indicates that gross volumetric changes are unlikely to account for the connectivity changes observable in the graph analysis between Time 1 and Time 2.

**Table 3:** White matter, gray matter, and cerebral spinal fluid volume in TBI and HC samples.

	TBI Time 1 Mean (sd)	TBI Time 2 Mean (sd)	HC Time 1 Mean (sd)	HC Time 2 Mean (sd)
**White matter volume (mm^3^)**	516.8 (56.0)	512.4 (54.3)	494.6 (67.0)	502.3 (66.2)
**Gray matter volume (mm^3^)**	649.3 (68.8)	647.0 (67.5)	640.6 (102.9)	654.3 (91.33)
**Cerebral spinal fluid (mm^3^)**	247.0 (46.3)	250.7 (49.0)	228.6 (30.1)	223.8 (21.3)

No significant between-group or between-time differences in tissue volume.

### Graph Theoretical Results: Global graph metrics

The data in Table 4 provide the global graph metrics for each group and time point. The data generally support a hyperconnectivity hypothesis during this early window after injury characterized by increased number and strength of network links globally (connectivity and clustering values non-significantly greater in TBI compared to HCs). Primary graph measures include 1) network strength (sum of weights over all edges in the graph), 2) number of network links, 3) path length (unweighted), 4) clustering coefficient, 5) small worldness (clustering/path length). The mean correlation coefficient across the graph was also computed. Two-tailed independent sample t-tests revealed significant or near-significant between-group differences at Time 1 for network strength (p = 0.05), number of links (p = 0.046), mean correlation coefficient between nodes (p = 0.04), clustering coefficient in the TBI sample (p = 0.06) and small worldness (clustering/path length) (p = 0.062). While the TBI retained relatively higher values for all indices at Time 2, the differences were not statistically significant and there were no between group differences, nor was there an effect of time on TBI connectivity. The data revealed comparable path length at both time points when comparing the two samples. The hyperconnectivity observed in global metrics is interpreted as a broad indicator of effects occurring at the local level as opposed to a global increase in connectivity (see below). The result of these regional increases in connectivity on global connectivity indices is modest (¾ to 1 standard deviation difference between groups across metrics).

**Table 4:** General graph properties in TBI and HC groups.

	TBI Sample Time 1 Mean(sd) n = 21	TBI Sample Time 2 Mean(sd) n = 21	TBI Combined Mean (sd) n = 21	Healthy Control Time 1 Mean (sd) n = 15	Healthy Control Time 2 Mean (sd) n = 15	Healthy Control Combined Mean (sd) n = 15
**Total Number of Connections**	478.67** 227.19	475.95 246.99	**477.31 153.20**	380.93** 128.53	409.9018 2.74	**395.42 134.45**
**Total Strength of Connections**	547.09** 192.1	539.90 204.92	**543.50 129.74**	465.40** 122.18	483.13 169.66	**474.27 126.63**
**Average path length**	1.60 0.19	1.61 0.21	**1.609 0.14**	1.67 0.14	1.669 0.185	**1.67 0.146**
**Clustering coefficient (weighted)**	0.2439* 0.08	0.248 0.085	0**.246 0.052**	0.213* 0.048	0.226 0.065	**0.220 0.048**

Global graph metrics. Significant between-group differences at Time 1(** p<0.05; *p<0.10). Note: **no** significant results survive corrections for multiple comparisons for an alpha of 0.05.

### Graph Theoretical Results: Degree distribution

**Figure 3:**
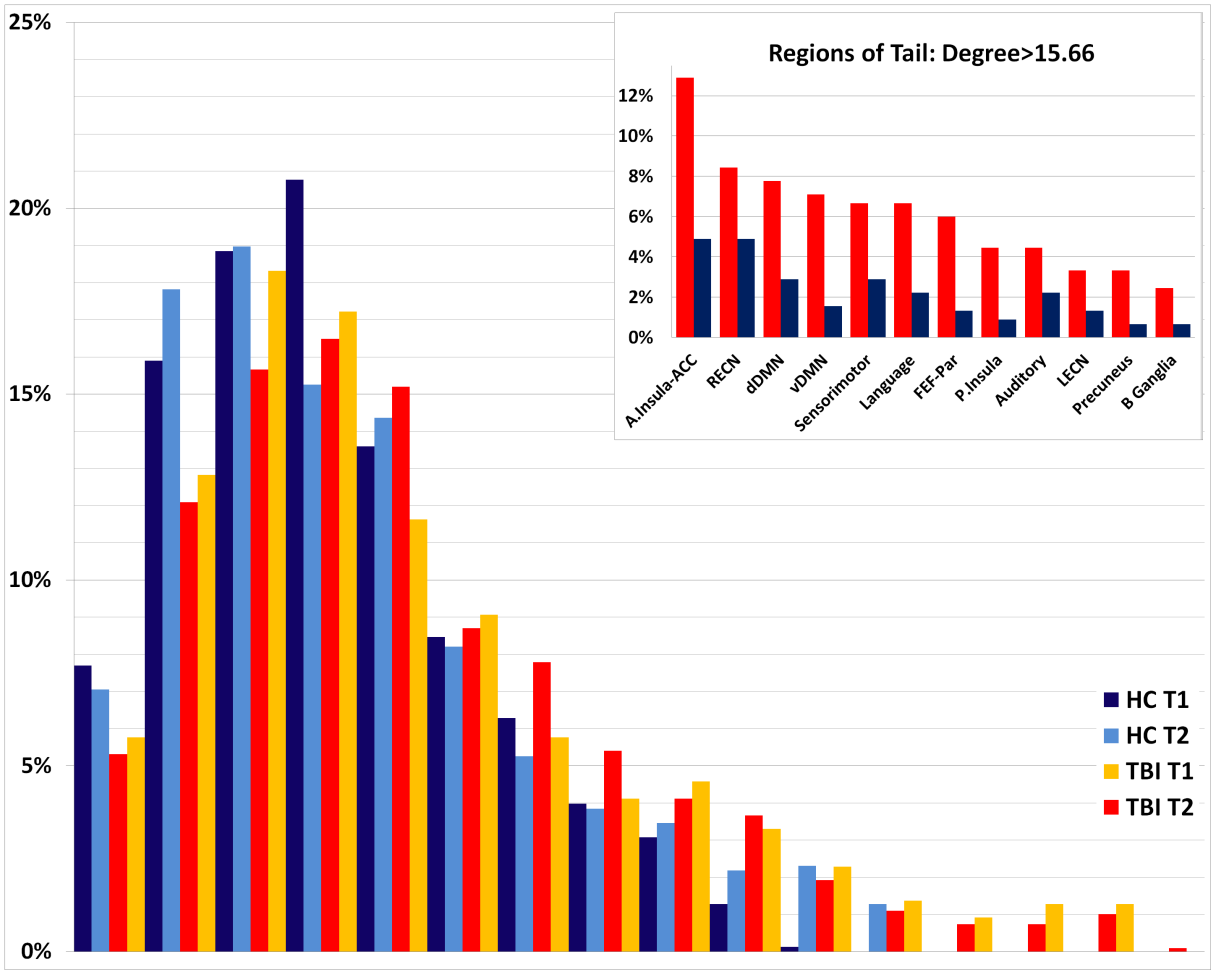
Probability distribution for TBI and HC groups at separate time points. Degree distributions for healthy control and TBI samples. Node degree (k), calculated as the sum of the weights on edges incident to a given node, is plotted against the fraction of nodes having given degree P(k), for each group at each time point. Values binned at increments of 2. **Inset**: the frequency of component members appearing in the heavy tail of p(k), or the most highly connected nodes. A-insula-ACC = anterior insula-anterior cingulate cortex (anterior salience network); dDMN = posterior cingulate to medial frontal (dorsal default mode network); LECN = Left dorsolateral prefrontal cortex and parietal (executive control network; P-insula = posterior insula (Salience Network); Par-FEF: Intraparietal Sulcus/Frontal Eye Fields (Visuospatial Network); RECN = right dorsolateral prefrontal cortex and parietal (executive control network); vDMN = Retrosplenial Cortex/Medial Temporal Lobe (Ventral Default Mode Network); B.Ganglia = basal ganglia. ***Note***: inset is collapsed to include all possible components assigned to each specific subnetwork and organized from highest to lowest node incidence in the TBI sample.

We examined the degree distribution for the entire sample (n = 36) for two reasons. First, we aimed to determine if the heavy tail that is a defining characteristic in power-law distributions was evident in the current network data. Second, we aimed to determine the components that comprised the most highly connected nodes within the distribution. In order to do so, a histogram of the degree distributions of all nodes for all subjects was plotted for both samples (TBI n = 1092; HC n = 780) at both time points. Here, node degree is plotted against the probability that a randomly selected node from given group at given time point has corresponding degree. Notice in Figure 3 that the degree distribution has the heavy right tail evident in the classic power-law degree distribution observed in many real-world networks, but drops-off in frequencies of very low degree nodes. The left side of the distribution has few very low-level connection prior to peaking and this can be attributed to the thresholding used to create the representative functional network from time series correlations. Specifically, pairwise correlations below the FDR threshold were discarded and did not appear as links in the graph. To determine the “hubs” of the graph, a lower bound for highly-connected regions was set at 2 sds above the mean for the Time 1 HC sample (degree = 15.66). The distribution reveals nodes of the highest degree are more likely to be observed in the TBI samples. For example, nodes with a degree of >15.66 make up 7.6% (119/1560) of all nodes HC sample and 15.1% (331/2184) of the nodes at in the TBI sample.

### Graph theoretical Results, most highly connected nodes

In order to examine the functional networks contributing to hyperconnectivity in TBI, we performed two separate calculations. First, we calculated the average degree for all nodes within each group and each time point and sorted the data based upon degree. Table 5 reveals the mean values for the most highly connected nodes that were at least 2 sds above the mean degree established at Time 1 in HCs. For Time 1, the TBI sample had significantly higher average nodal values (Time 1 mean = 9.19, sd = 1.07, se = 0.149) compared to the HC sample (Time 1 mean = 7.32, sd = 1.29, se = 0.179; t(102) = −7.98; p<0.001]. This finding was again observed at Time 2 (TBI mean = 9.13, sd = 1.074, se = 0.150; HC mean = 7.88, sd = 1.39, se = 0.194; t(102) = −6.92; p<0.001].

**Table 5:** Most highly connected nodes (hubs) for TBI and HCs at Time 1 and Time 2 (mean degree values).

TBI Time 1 (n = 21)		TBI Time 2 (n = 21)	
Mean Degree	Spatial Component (ID#)	Mean sum of links	Spatial Component
11.27	R ECN (34)	11.12	Language (46)
10.97	P. Salience (35)	11.03	Sensorimotor (15)
10.86	Language (41)	10.98	FEF-par (39)
10.86	vDMN (20)	10.63	PCC/MPFC (25)
10.55	A. Salience (51)	10.59	R ECN (45)
10.53	dDMN (25)	10.57	A. Salience (47)
10.43	dDMN (31)	10.43	R ECN (34)
10.40	Precuneus (49)	10.24	A. Salience (4)
10.35	Sensorimotor (15)	10.24	A. Salience (16)
10.27	A. Salience (48)	10.10	vDMN (20)
10.25	FEF-par (50)	10.06	dDMN (52)
10.12	Auditory (9)		
10.08	PCC/MPFC (30)		
10.06	Auditory (32)		
10.04	R ECN (45)		
10.03	A. Salience (42)		
9.92	Language (3)		

HC Time 1 (n = 15)		HC Time 2 (n = 15)	
Mean Degree	Spatial Component	Mean sum of links	Spatial Component
10.52	R.ECN (45)	11.06	R ECN (34)
		10.16	Auditory (32)

The most highly connected nodes determined by cutoff of 9.9 (2 standard deviations above the mean degree for HC data at Time 1). ***Note***: values for components listed at Time 1, not listed for Time 2 (41 = 9.72; 51 = 8.8; 31 =  9.54; 49 = 8.96; 48 = 9.70; 50 = 8.91; 9 = 9.3; 42 = 9.31; 3 = 9.83) and for Time 2 but not Time 1 (52 = 9.83; 4 = 9.42; 39 = 8.79; 47 = 8.69; 16 = 8.69) were also at least 1 sd above the HC Time 1 mean, but below 2 sd cutoff. Components in **bold** are identical components between time points.

**Figure 4:**
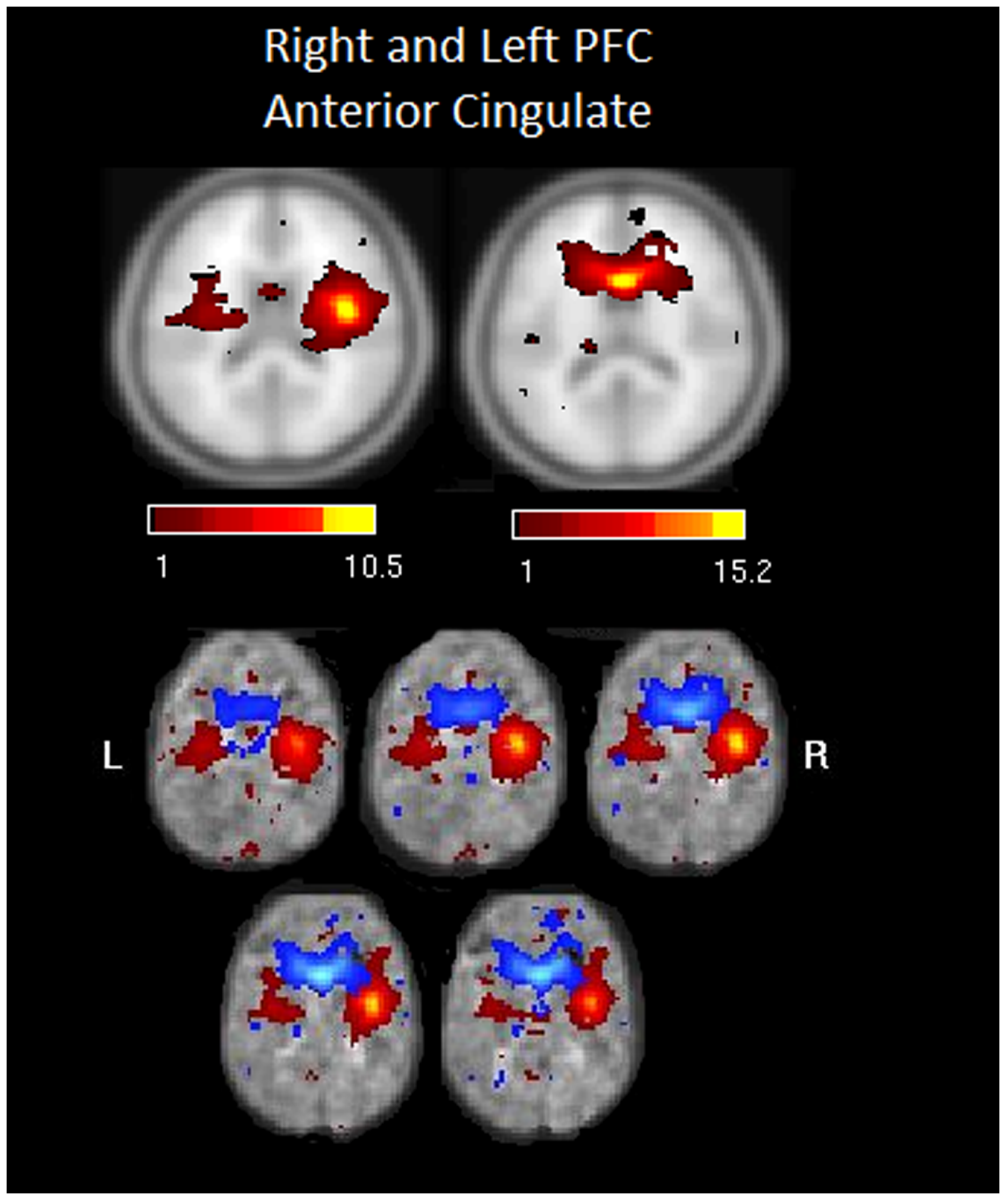
Illustrates the two of the most common nodes occurring for both samples at both time points for the right ECN network.

**Figure 5:**
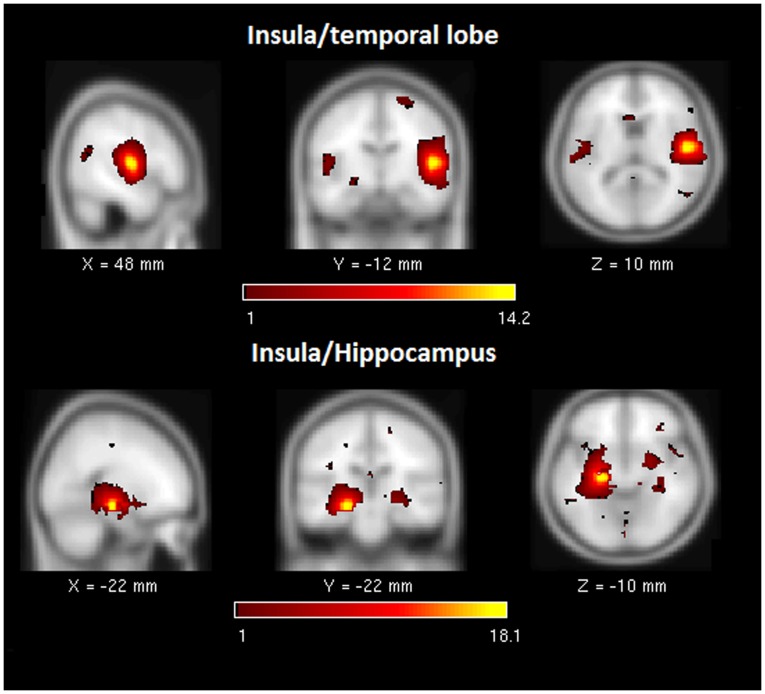
Illustrates two examples of functional components of the anterior insula (SN) in the “tail” of the degree distribution for the TBI sample. Note: SN = salience network.

**Figure 6:**
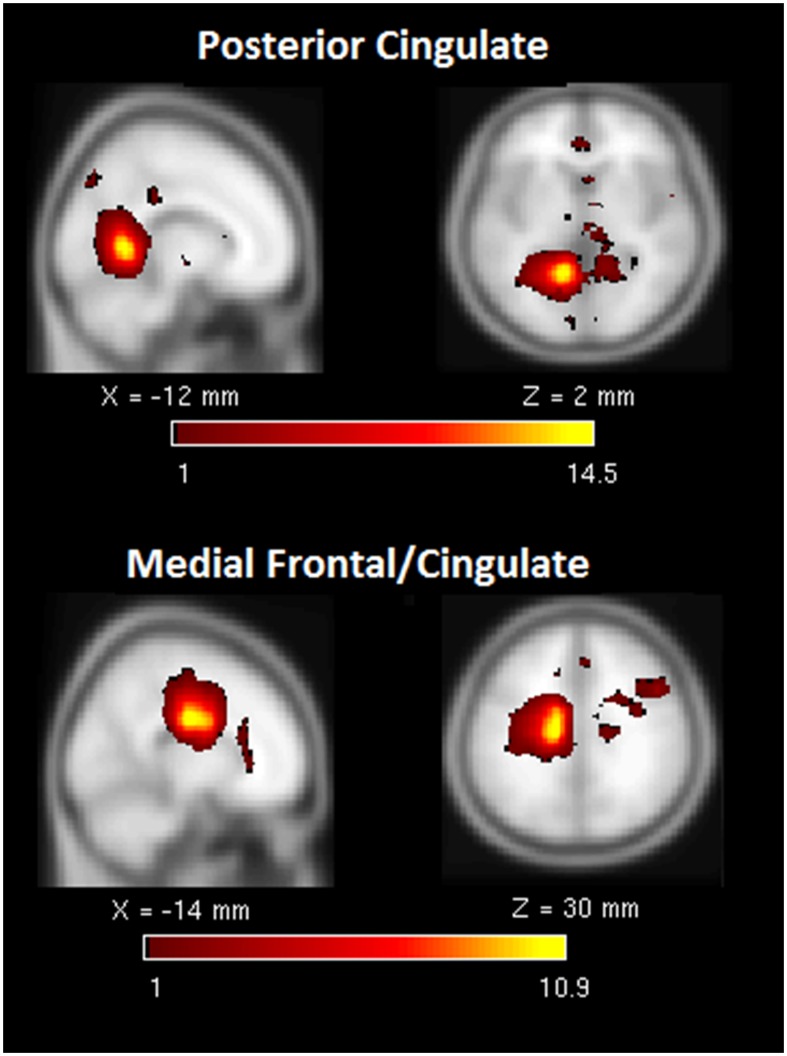
Illustrates two examples of functional components of the dorsal DMN in the “tail” of the degree distribution for the TBI sample. Note: DMN = default mode network, Med = Medial, PCC = posterior cingulate cortex.

Second, we calculated the frequency of components comprising the heavy tail in the probability distribution in Figure 3. The most frequently observed components for the TBI sample were the: 1) anterior insula-ACC (salience network), 2) right executive control network, 3) PCC to medial frontal (dorsal DMN) and 4) the retrosplenial cortex-medial temporal lobe (ventral DMN) (see Figure 3 inset). The connectivity in these core subnetworks in the TBI sample was not reflected in the HC sample, where there was a more even distribution of high-degree nodes across the classically recognized subnetworks in the brain with relative equally high connectivity in dDMN, sensorimotor, language and auditory networks. Figures 4–6 illustrate several of the most common components represented in both Table 5 and the distribution tail from Figure 3.

### Behavioral performance and Hubs

We examined the relationship between the cognitive tests showing the greatest difference between the TBI and HC samples (see Table 2) and the most highly connected subnetworks (i.e., components) appearing at both Time 1 and Time 2 in TBI. To do so, the average scores for three cognitive measures showing significant decrements in TBI at Time 1 were computed: 1) Stroop Color-word, 2) VSAT total score, and 3) Trails B. These three cognitive measures were correlated with the components most commonly appearing in Table 5 and the members of the distribution “tail” (Figure 3 Inset). These three components included the ECN (Figure 4), A. Insula (Figure 5), and the DMN (Figure 6). The results reveal low to moderate correlation values (Stroop x RECN Time 2: r = 0.289; Trails B x RECN Time 1, r = −0.37; Trails B x A.Insula Time 2, r = 0.437) tha*t did not survive correction for multiple comparisons (3 cognitive tests x 3 components at 2 time points.* The results indicate that the relationships between hub connectivity and cognitive efficiency are likely subtle and dependent upon multiple subject and network factors for expression including network context (i.e., timing, inclusion of additional connections).

## Discussion

We used functional MRI and graph theory methods to examine whole brain connectivity changes early after moderate and severe TBI. Using a weighted graph we were able to track not only the influence of TBI on the number of connections but also their strength, which is a decided advantage to this approach. The primary hypothesis that brain connectivity will increase after TBI was generally supported; global metrics showed greater connectivity in the TBI sample and targeted analysis revealed specific nodes where hyperconnectivity is occurring after injury. This effect was evident when examining the mean degree for the 52 nodes, there was significantly greater connectivity in the TBI compared to HC sample. The between-group differences in global graph characteristics such as mean degree and mean number of links were modest (Table 4), and this finding is not unexpected given that we do not anticipate that all network nodes are significantly increasing during recovery. Instead, the highest degree nodes were selectively observed in several core subnetworks such as the SN, ECN, and parts of the DMN. The primary implication for these data is that physical disruption of networks results in an increase in connectivity in select nodes (see inset to Figure 3). The most impressive evidence for this is Figure 3, where the heavy power law tail is disproportionately composed of nodes coming from TBI cases.

The hyperconnectivity hypothesis proposed here is generally consistent with a greater literature, although some qualification is required. Recently in a cross-sectional study of TBI, Pandit and colleagues [13] found *diminished* connectivity in critical nodes and loss of small-worldness. These findings are not consistent with the results here and there are likely several reasons for this. First, the networks in Pandit et al. and the current work are quite different with respect to the scale of the networks investigated (15 vs. 52 nodes) and, therefore, the data in Pandit and colleagues may be capturing local effects or those within a relatively constrained set of subnetworks. As we elaborate upon below, hyperconnectivity is not uniformly expressed with examples of connectivity loss even within core subnetworks like the DMN. Second, the work by Pandit and colleagues examined chronic TBI, most greater than 2 years post injury, where we might expect to see maturation of the effects observable in the early sample presented here and possibly even connectivity loss in the case of older subjects (age ranges in that study from 18–54). Finally, the current approach uses a weighted as opposed to a binary network providing a richer representation of pairwise regional communication and sensitivity to changing connection strength. In general, the preponderance of this literature has established connectivity increases after moderate and severe injury (11–12, 14, 25, 37, 88] and the current data demonstrate that this general response is evident within the first few months after injury.

### Connectivity in hubs after injury: the rich do get richer

It was also a goal to determine if core subnetworks disproportionately accounted for changing connectivity after TBI. We hypothesized that hyperconnectivity would be expressed in three large-scale subnetworks: the SN, ECN, and DMN. This hypothesis was generally supported in several different ways. First, when examining the subnetworks contributing to the heavy tail of the probability distribution, the subnetworks appearing with the highest frequency was the anterior insula of the SN and the ventral and dorsal DMN, which is consistent with two separate findings in the TBI literature. There is now a growing body of literature showing enhanced DMN connectivity after TBI [17], [37]–[38], [88]–[89]. The role of hyperconnectivity in the DMN after TBI remains uncertain, with several possible explanations, including a failure to deactivate the DMN as a source of interference during goal-directed behavior [17]. While this explanation has intuitive appeal, it may be the case that a hyperconnectivity response both for goal-directed and internal-state networks observed here poses a challenge to seamless transition between these networks. Second, the finding that the anterior insular salience network may be heightened in brain injury has received recent support both in longitudinal [37] and cross-sectional studies [38] and even in a study of the minimally conscious state [90]. This finding indicates that of the most highly connected nodes (i.e., the tail of the probability distribution), the anterior salience network, including insula and anterior cingulate cortex, accounted for the highest percentage of observations. Enhanced involvement of the salience network has been interpreted elsewhere as providing attentional control and operating as a conduit between internal brain states and external stimulation [91]. Finally, when examining the nodes with highest average degree, multiple components within the right ECN network showed degree >2sds above the HC mean in the TBI sample and the RECN was the second most frequent component observed in the heavy tail of the degree distribution. This finding is consistent with a literature emphasizing the role of the *right* prefrontal cortex in processing novelty [92]–[93] and increased task load associated with neurological insult, including TBI [33], [70]. Neurological insult creates an environment of reduced automaticity and increased supervisory demand for all levels of information processing and the mean connectivity observable in the right ECN may play a role in this. Overall, the observation that “the rich get richer” is consistent with a preferential attachment model of growth in scale free networks, where new connections are more likely to be collected by the most connected nodes [94].

It should be emphasized that the hyperconnectivity observed after injury may be dissociable within networks; while nodes within the DMN and SN were the most commonly represented in network hubs (or distribution “tail”), decreased involvement within these networks was also observed. For example, there were also components within the ventral and dorsal DMN that showed connectivity decline over time in the TBI sample. This observation reveals the functional complexity of nodes within the DMN which more recent work has demonstrated to have divergent roles [95]–[96]. While the ICA used here aids in determining the functional signatures (components) that are spatially consistent with the DMN, this approach also permits differential analysis of how the distinct connectivity between these signatures are interacting both within and outside the DMN.

### Connectivity and clinical and demographic factors

In any study of TBI there is inherent concern about heterogeneity in the nature and severity of the injury between the individuals comprising any group. Although this issue is certainly not unique to TBI, it is a concern universally echoed in this clinical sample. The current study holds significant control in one area often not considered: time post injury. In addition to the opportunity to examine network change during a critical recovery window, examining all individuals at 3 and 6 months post PTA resolution provides some equilibration for neurological status at the first measurement time point. However, several other critical factors require consideration to provide context for the current findings. First, while the location of injury and severity of injury are difficult to maintain constant in TBI work; it should be noted that there was no obvious relationship between injury factors and graph metrics in this sample. For example, when splitting the results based upon greater and lesser network degree, the TBI subgroups were equally likely to show evidence of diffuse injuries indicative of DAI, focal injury (subdural hematoma/contusion), or mixed injuries. This is entirely consistent with the signal amplitude literature in fMRI whereby pathophysiology was not a determinant of neural recruitment [33]–[34]. With respect to injury severity, GCS score was a poor predictor of connectivity indices (i.e., degree and number of connections), so gross indicators of injury severity may not reliably predict global brain response. While the age range in this sample is comparable to most of the connectivity work conducted in TBI to date [13]–[14], [17], [37], it was quite broad, including young adults to middle-aged participants (ages 18–53). Increasingly nuanced investigation of brain connectivity following TBI will likely reveal potentially important differences in the brain response across the developmental spectrum. For example, it may be the case that a common response to injury is hyperconnectivity but the degree to which this is expressed may be moderated by age or resource availability (for a critical review see [19]). Even given these considerations, it appears that increased connectivity in critical network nodes is a robust effect emerging even in the context of distinct injury severity and location of pathology.

While capturing the various elements of clinical recovery after TBI is difficult to do with a battery of cognitive tests, we have some indication of behavioral improvement between Time 1 and Time 2. Measures of connectivity, however, did not appear to mirror these changes in any straightforward way. When using the behavioral data to provide context for these imaging findings, the network x behavioral findings are low to modest (accounting for ∼10–20% of the total variance). These relationships may indeed require more nuanced analysis including examining the specific clinical presentations associated with connectivity. It will also be important to model nonlinear relationships or examine “windows of benefit” for hyperconnectivity. As these relationships are established, network connectivity may begin to enhance our understanding of brain plasticity and its role in clinical recovery.

### Summary and Future Directions

The findings here provide evidence that a common network response to traumatic brain injury is hyperconnectivity, observable over the course of the first six months of recovery in this sample of moderate and severe TBI. There is also evidence that hyperconnectivity may be differentially observed in the “rich club” or nodes that form the backbone to information transfer in the brain. However, there are several areas for study refinement and future work to continue to clarify the meaning of hyperconnectivity after neurological disruption. While the sample heterogeneity here matches the literature, there may be some subtle but important influences of age on connectivity result. Similarly, if the use of more than one MRI scanner added error to the data, it would reduce the sensitivity to detect subtle effects in connectivity. With regard to MRI facility, analysis of data from separate facilities revealed no systematic bias (e.g., data from one MRI machine was no more likely to reveal increased/decreased connectivity in the TBI sample). In addition, the time series data used here are appropriately long for these analyses, but the approach does assume stationarity within each time series. While this approach is fine for our purposes of examining large-scale network change, for a nuanced understanding of the network changes including network modularity and flexibility, within-time-point analyses are required. There are also questions to be answered regarding the how the onset of goal-directed behavior (i.e., task) influences global and local connectivity. Overall, it should be a goal of future work to establish those contextual demands giving rise to hyperconnectivity and the physical resource thresholds that permit its expression.

## Supporting Information

Table S1 **General graph properties in TBI and HC using an inclusive graph (components: 60).** The current table includes “equivocal” components to demonstrate the robustness of the primary findings in Table 3. The TBI sample shows greater, but non-significant increases in global metrics of connectivity (*p<.0.10 during independent samples one-tailed test). **Note**: no significant results survive corrections for multiple comparisons at alpha = 0.05.
(DOC)

Table S2 **General graph properties in TBI and HC in Sparse Graph (minimum r-value = 0.403).** Metrics for the TBI sample show greater, but non-significant increases in global metrics of connectivity (*p<.0.10; independent samples one-tailed test). **Note**: no significant results survive corrections for multiple comparisons at alpha = 0.05.
(DOC)

## References

[1] Cayley A (1875) Ueber due Analytischen Figuren, welche in der Mathematik Baume genannt werden und ihre Anwendung auf die Theorie chemischer Verbindungen. Berichte der deutschen Chemischen Gesellschaft. 8: 1056–1059.

[2] Sylvester JJ (1878) Chemistry and Algebra. Nature. 17: 284.

[3] Scott JP (2000) Social Network Analysis: A Handbook, Vol., Sage Publications, Thousand Oaks, CA.

[4] Hilgetag CC, Burns GA, O'Neill MA, Scannell JW, Young MP (2000) Anatomical connectivity defines the organization of clusters of cortical areas in the macaque monkey and the cat. *Philos Trans R Soc Lond B Biol Sci*. 355: 91–110. 10703046 10.1098/rstb.2000.0551 PMC1692723

[5] Micheloyannis S, Pachou E, Stam CJ, Vourkas M, Erimaki S, et al (2006) Using graph theoretical analysis of multi channel EEG to evaluate the neural efficiency hypothesis. Neurosci Lett. 402: 273–7. 16678344 10.1016/j.neulet.2006.04.006

[6] Stam CJ (2004) Functional connectivity patterns of human magnetoencephalographic recordings: a ‘small-world’ network? Neurosci Lett. 355: 25–8. 14729226 10.1016/j.neulet.2003.10.063

[7] Stam CJ, Jones BF, Nolte G, Breakspear M, Scheltens P (2007) Small-world networks and functional connectivity in Alzheimer's disease. Cereb Cortex. 17: 92–9. 16452642 10.1093/cercor/bhj127

[8] Bullmore E, Sporns O (2009) Complex brain networks: graph theoretical analysis of structural and functional systems. Nat Rev Neurosci. 10: 186–98. 19190637 10.1038/nrn2575

[9] Sheline YI, Raichle ME (2013) Resting state functional connectivity in preclinical Alzheimer's disease. *Biol Psychiatry* 74: 340–7. 23290495 10.1016/j.biopsych.2012.11.028 PMC3537262

[10] Castellanos NP, Paúl N, Ordóñez VE, Demuynck O, Bajo R, et al (2010) Reorganization of functional connectivity as a correlate of cognitive recovery in acquired brain injury. *Brain* 133: 2365–81. 20826433 10.1093/brain/awq174

[11] Castellanos NP, Leyva I, Buldú JM, Bajo R, Paúl N, et al (2011) Principles of recovery from traumatic brain injury: reorganization of functional networks. *Neuroimage* 55: 1189–99. 21195199 10.1016/j.neuroimage.2010.12.046

[12] Nakamura T, Hillary FG, Biswal BB (2009) Resting network plasticity following brain injury. *PLoS One* 4: e8220. 20011533 10.1371/journal.pone.0008220 PMC2788622

[13] Pandit AS, Expert P, Lambiotte R, Bonnelle V, Leech R, et al (2013) Traumatic brain injury impairs small-world topology. *Neurology* 80: 1826–33. 23596068 10.1212/WNL.0b013e3182929f38 PMC3908350

[14] Caeyenberghs K, Leemans A, Heitger MH, Leunissen I, Dhollander T, et al (2012) Graph analysis of functional brain networks for cognitive control of action in traumatic brain injury. *Brain* 135(Pt 4): 1293–307. 22427332 10.1093/brain/aws048

[15] Vakhtin A, Calhoun VD, Jung RE, Prestopnik JL, Taylor PA, et al (2013) Changes in Intrinsic Functional Brain Networks Following Blast-Induced Mild Traumatic Brain Injury. *Brain Injury* 27(11): 1304–10. 24020442 10.3109/02699052.2013.823561 PMC5075489

[16] Bonnelle V, Leech R, Kinnunen KM, Ham TE, Beckmann CF, et al (2011) Default mode network connectivity predicts sustained attention deficits after traumatic brain injury. *J Neurosci* 31: 13442–51. 21940437 10.1523/JNEUROSCI.1163-11.2011 PMC6623308

[17] Sharp DJ, Beckmann CF, Greenwood R, Kinnunen KM, Bonnelle V, et al (2011) Default mode network functional and structural connectivity after traumatic brain injury. *Brain* 134: 2233–47. 21841202 10.1093/brain/awr175

[18] Hillary FG, Medaglia JD, Gate K, Molenaar PMC, Slocomb J, et al (2011) Examining working memory task acquisition in a disrupted neural network. *Brain*. 134(Pt 5): 1555–70. 21571783 10.1093/brain/awr043

[19] Hillary FG, Roman C, Venkatesan U, Rajtmajer SM, Bajo R, et al. (2014) Hyperconnectivity as a fundamental response to neurological disruption. N*europsychology*. June, Epub ahead of print. PMID: 24933491 10.1037/neu0000110 24933491

[20] Achard S, Salvador R, Whitcher B, Suckling J, Bullmore E (2006) A resilient, low-frequency, small-world human brain functional network with highly connected association cortical hubs. J Neurosci. 26: 63–72. 16399673 10.1523/JNEUROSCI.3874-05.2006 PMC6674299

[21] Albert R, Jeong H, Barabasi AL (2000) Error and attack tolerance of complex networks. *Nature* 406: 378–82. 10935628 10.1038/35019019

[22] Harriger L, van den Heuvel MP, Sporns O (2012) Rich club organization of macaque cerebral cortex and its role in network communication. *PLoS One* 7: e46497. 23029538 10.1371/journal.pone.0046497 PMC3460908

[23] van den Heuvel MP, Kahn RS, Goñi J, Sporns O (2012) High-cost, high-capacity backbone for global brain communication. Proc Natl Acad Sci U S A. 109: 11372–7. 22711833 10.1073/pnas.1203593109 PMC3396547

[24] Sporns O (2011) The human connectome: a complex network. Ann NY Acad Sci. 1224: 109–25. 21251014 10.1111/j.1749-6632.2010.05888.x

[25] Caeyenberghs K, Leemans A, Leunissen I, Michiels K, Swinnen SP (2013) Topological correlations of structural and functional networks in patients with traumatic brain injury. *Front Hum Neurosci*. 7: 726. 24204337 10.3389/fnhum.2013.00726 PMC3817367

[26] Vul E, Pashler H (2012) Voodoo and circularity errors. *Neuroimage* 62: 945–8. 22270348 10.1016/j.neuroimage.2012.01.027

[27] Calhoun VD, Adali T, Pearlson GD, Pekar JJ (2001) Spatial and temporal independent component analysis of functional MRI data containing a pair of task-related waveforms. *Hum Brain Mapp* 13: 43–53. 11284046 10.1002/hbm.1024 PMC6871956

[28] Calhoun VD, Liu J, Adali T (2009) A review of group ICA for fMRI data and ICA for joint inference of imaging, genetic, and ERP data. Neuroimage. 45: S163–72. 19059344 10.1016/j.neuroimage.2008.10.057 PMC2651152

[29] Xu J, Zhang S, Calhoun VD, Monterosso J, Li CS, et al (2013) Task-related concurrent but opposite modulations of overlapping functional networks as revealed by spatial ICA. *Neuroimage* 79: 62–71. 23611864 10.1016/j.neuroimage.2013.04.038 PMC3677796

[30] Hillary FG, Biswal BB (2007) The influence of neuropathology on the FMRI signal: a measurement of brain or vein? Clin Neuropsychol. 21(1): 58–72. 17366278 10.1080/13854040601064542

[31] Pouratian N, Sheth S, Bookheimer SY, Martin NA, Toga AW (2003) Applications and limitations of perfusion-dependent functional brain mapping for neurosurgical guidance. *Neurosurg Focus*. 15: E2. 10.3171/foc.2003.15.1.2 15355004

[32] Strigel RM, Moritz CH, Haughton VM, Badie B, Field A, et al (2005) Evaluation of a signal intensity mask in the interpretation of functional MR imaging activation maps. AJNR Am J Neuroradiol. 26: 578–84. 15760869 PMC7976496

[33] Hillary FG, Genova HM, Chiaravalloti ND, Rypma B, DeLuca J (2006) Prefrontal modulation of working memory performance in brain injury and disease. Hum Brain Mapp. 27(11): 837–47. 16447183 10.1002/hbm.20226 PMC6871387

[34] Hillary FG (2008) Neuroimaging of working memory dysfunction and the dilemma with brain reorganization hypotheses. *J Int Neuropsychol Soc*. 14: 526–34. 18577281 10.1017/S1355617708080788

[35] Turner GR, McIntosh AR, Levine B (2011) Prefrontal Compensatory Engagement in TBI is due to Altered Functional Engagement Of Existing Networks and not Functional Reorganization. Front Syst Neurosci. 5: 9. 21412403 10.3389/fnsys.2011.00009 PMC3048219

[36] Leech R, Braga R, Sharp DJ (2012) Echoes of the brain within the posterior cingulate cortex. J Neurosci. 32: 215–22. 22219283 10.1523/JNEUROSCI.3689-11.2012 PMC6621313

[37] Bonnelle V, Ham TE, Leech R, Kinnunen KM, Mehta MA, et al (2012) Salience network integrity predicts default mode network function after traumatic brain injury. *Proc Natl Acad Sci U S A.* 109: 4690–5. 22393019 10.1073/pnas.1113455109 PMC3311356

[38] Hillary FG, Slocomb J, Hills EC, Fitzpatrick NM, Wang J, et al (2011) Changes in resting connectivity during recovery from severe traumatic brain injury. *Int J Psychophysiology* 82: 115–23. 10.1016/j.ijpsycho.2011.03.011 21473890

[39] Sidlauskaite J, Wiersema JR, Roeyers H, Krebs RM, Vassena E, et al. (2014) Anticipatory processes in brain state switching - Evidence from a novel cued-switching task implicating default mode and salience networks. *Neuroimage*. May 12 pii: S1053-8119(14)00375-9. 10.1016/j.neuroimage.2014.05.010 24830839

[40] DeLuca J, Schultheis MT, Madigan NK, Christodoulou C, Averill A (2000) Acquisition versus retrieval deficits in traumatic brain injury: implications for memory rehabilitation. *Arch Phys Med Rehabil.* 81: 1327–33. 11030497 10.1053/apmr.2000.9390

[41] McDowell S, Whyte J, D'Esposito M (1997) Working memory impairments in traumatic brain injury: evidence from a dual-task paradigm. Neuropsychologia 35(10) p 1341–53. 9347480 10.1016/s0028-3932(97)00082-1

[42] Mazaika P, Hoeft F, Glover GH, Reiss AL (2009) Software for fMRI Analysis for Clinical Subjects. *Human Brain Mapping*.

[43] Bouilleret V, Cardamone L, Liu YR, Fang K, Myers DE, et al (2009) Progressive brain changes on serial manganese-enhanced MRI following traumatic brain injury in the rat. *J Neurotrauma* 26: 1999–2013. 19604101 10.1089/neu.2009.0943

[44] Jones NC, Cardamone L, Williams JP, Salzberg MR, Myers D, et al (2008) Experimental traumatic brain injury induces a pervasive hyperanxious phenotype in rats. *J Neurotrauma*. 25: 1367–74. 19061380 10.1089/neu.2008.0641

[45] Liu YR, Cardamone L, Hogan RE, Gregoire MC, Williams JP, et al (2010) Progressive metabolic and structural cerebral perturbations after traumatic brain injury: an in vivo imaging study in the rat. *J Nucl Med.* 51: 1788–95. 21051651 10.2967/jnumed.110.078626

[46] Shimamura M, Garcia JM, Prough DS, Dewitt DS, Uchida T, et al (2005) Analysis of long-term gene expression in neurons of the hippocampal subfields following traumatic brain injury in rats. *Neuroscience*. 131: 87–97. 15680694 10.1016/j.neuroscience.2004.10.041

[47] Shultz SR, Cardamone L, Liu YR, Hogan RE, Maccotta L, et al (2013) Can structural or functional changes following traumatic brain injury in the rat predict epileptic outcome? *Epilepsia*. 54: 1240–50. 23718645 10.1111/epi.12223 PMC4032369

[48] Stibick DL, Feeney DM (2001) Enduring vulnerability to transient reinstatement of hemiplegia by prazosin after traumatic brain injury. J Neurotrauma. 18: 303–12. 11284550 10.1089/08977150151070955

[49] English SW, Turgeon AF, Owen E, Doucette S, Pagliarello G, et al (2013) Protocol management of severe traumatic brain injury in intensive care units: a systematic review. *Neurocrit Care*. 18: 131–42. 22890909 10.1007/s12028-012-9748-3

[50] Jimenez N, Ebel BE, Wang J, Koepsell TD, Jaffe KM, et al (2013) Disparities in disability after traumatic brain injury among Hispanic children and adolescents. *Pediatrics*. 131: e1850–6. 23650302 10.1542/peds.2012-3354 PMC3666112

[51] Jourdan C, Bosserelle V, Azerad S, Ghout I, Bayen E, et al (2013) Predictive factors for 1-year outcome of a cohort of patients with severe traumatic brain injury (TBI): results from the PariS-TBI study. *Brain Inj.* 27: 1000–7. 23730948 10.3109/02699052.2013.794971

[52] Kouloulas EJ, Papadeas AG, Michail X, Sakas DE, Boviatsis EJ (2013) Prognostic value of time-related Glasgow Coma Scale components in severe traumatic brain injury: a prospective evaluation with respect to 1-year survival and functional outcome. *Int J Rehabil Res*. 36: 260–7. 23470551 10.1097/MRR.0b013e32835fd99a

[53] McCauley SR, Wilde EA, Moretti P, Macleod MC, Pedroza C, et al (2013) Neurological Outcome Scale for Traumatic Brain Injury: III. Criterion-Related Validity and Sensitivity to Change in the NABIS Hypothermia-II Clinical Trial. *J Neurotrauma*. 30: 1506–11. 23617608 10.1089/neu.2013.2925 PMC3751279

[54] Radford K, Phillips J, Drummond A, Sach T, Walker M, et al. (2013) Return to work after traumatic brain injury: cohort comparison and economic evaluation. *Brain Inj.* 27: , 507–20. 10.3109/02699052.2013.766929 23473058

[55] Walker WC, Marwitz JH, Wilk AR, Ketchum JM, Hoffman JM, et al. (2013) Prediction of headache severity (density and functional impact) after traumatic brain injury: A longitudinal multicenter study. Cephalalgia. 33: , 998–1008. 10.1177/0333102413482197 23575819

[56] Hillary FG, Genova HM, Medaglia JD, Fitzpatrick NM, Chiou KS, et al. (2010)The nature of processing speed deficits in traumatic brain injury: is less brain more? *Brain Imaging Behav* 4(2) : p. 141–54.xz 10.1007/s11682-010-9094-z 20502993

[57] Madigan NK, DeLuca J, Diamond BJ, Tramontano G, Averill A (2000) Speed of information processing in traumatic brain injury: modality-specific factors. *J Head Trauma Rehabil* 15: , 943–56. 10.1097/00001199-200006000-00007 10785624

[58] McAllister TW, Flashman LA, McDonald BC, Saykin AJ (2006) Mechanisms of working memory dysfunction after mild and moderate TBI: evidence from functional MRI and neurogenetics. *J Neurotrauma* 23(10): 1450–67. 17020482 10.1089/neu.2006.23.1450

[59] Trenerry MR, Crosson B, DeBoe J, Leber WR (1989) Professional manual: visual search and attention test. Psychological Assessment Resources, Lutz.

[60] Jensen AR, Rohwer WD (1966) The stroop color-word test: A review. *Acta Psychologica*. 25: 36–93. 5328883 10.1016/0001-6918(66)90004-7

[61] Stroop JR (1935) Studies of interference in serial verbal reactions. Journal of Experimental Psychology. 18: 643–662.

[62] Army Individual Test Battery (1990) Manual of Directions and Scoring. Washington, DC: War Department, Adjutant General's Office.

[63] Reitan RM, Wolfson D (1985) The halstead-reitan neuropsychological test battery: Therapy and clinical interpretation. Tucson, AZ: Neuropsychological Press.

[64] Wechsler D (1997) Wechsler Adult Intelligence Scale - Third Edition. Administration and Scoring Manual. The Psychological Corporation, San Antonio.

[65] Benedict RH, Duquin JA, Jurgensen S, Rudick RA, Feitcher J, et al (2008) Repeated assessment of neuropsychological deficits in multiple sclerosis using the Symbol Digit Modalities Test and the MS Neuropsychological Screening Questionnaire. *Mult Scler.* 14: 940–6. 18573822 10.1177/1352458508090923

[66] Büki A, Povlishock JT (2006) All roads lead to disconnection? – Traumatic axonal injury revisited. *Acta Neurochirurgica.* 148: 181–194. 16362181 10.1007/s00701-005-0674-4

[67] Fujiwara E, Schwartz ML, Gao F, Black SE, Levine B (2008) Ventral frontal cortex functions and quantified MRI in traumatic brain injury. *Neuropsychologia*. 46: 461–74. 17976665 10.1016/j.neuropsychologia.2007.08.027 PMC2287189

[68] Wu HM, Huang SC, Hattori N, Glenn TC, Vespa PM, et al (2004) Subcortical white matter metabolic changes remote from focal hemorrhagic lesions suggest diffuse injury after human traumatic brain injury. *Neurosurgery*. 55: 1306–15 discussion 1316–7. 15574212 10.1227/01.neu.0000143028.08719.42

[69] Kirchner WK (1958) Age differences in short-term retention of rapidly changing information. J *Exp Psychol* 55: 352–8. 13539317 10.1037/h0043688

[70] Medaglia JD, Chiou KS, Slocomb J, Fitzpatrick NM, Wardecker BM, et al (2012) The less BOLD, the wiser: support for the latent resource hypothesis after traumatic brain injury. *Hum Brain Mapp.* 33: 979–93. 21591026 10.1002/hbm.21264 PMC6870270

[71] Abou-Elseoud A, Starck T, Remes J, Nikkinen J, Tervonen O, et al (2010) The effect of model order selection in group PICA. *Hum Brain Mapp*. 31: 1207–16. 20063361 10.1002/hbm.20929 PMC6871136

[72] Allen EA, Erhardt EB, Damaraju E, Gruner W, Segall JM, et al (2011) A baseline for the multivariate comparison of resting-state networks. *Front Syst Neurosci.* 5: 2. 21442040 10.3389/fnsys.2011.00002 PMC3051178

[73] Kiviniemi V, Starck T, Remes J, Long X, Nikkinen J, et al (2009) Functional segmentation of the brain cortex using high model order group PICA. *Hum Brain Mapp* 30: 3865–86. 19507160 10.1002/hbm.20813 PMC6870574

[74] Smith SM, Fox PT, Miller KL, Glahn DC, Fox PM, et al (2009) Correspondence of the brain's functional architecture during activation and rest. *Proc Natl Acad Sci* U S A. 106: 13040–5. 19620724 10.1073/pnas.0905267106 PMC2722273

[75] Ystad MA, Lundervold AJ, Wehling E, Espeseth T, Rootwelt H, et al (2009) Hippocampal volumes are important predictors for memory function in elderly women. *BMC Med Imaging.* 9: 17. 19698138 10.1186/1471-2342-9-17 PMC2743662

[76] Allen EA, Erhardt E, Wei Y, Eichele T, Calhoun VD (2012) Capturing inter-subject variability with group independent component analysis of fMRI data: a simulation study. *NeuroImage* vol. 59 pp 4141–4159. 22019879 10.1016/j.neuroimage.2011.10.010 PMC3690335

[77] Calhoun VD, Adali T, Pearlson GD, Pekar JJ (2001) A method for making group inferences from functional MRI data using independent component analysis. Hum Brain Mapp. 14: 140–51. 11559959 10.1002/hbm.1048 PMC6871952

[78] Du Y, Allen EA, He H, Sui J, Calhoun VD (2014) Comparison of ICA based fMRI artifact removal: single subject and group approaches, in Proceedings of the Organization of Human Brain Mapping, Hamburg, Germany.

[79] Erhardt EB, Rachakonda S, Bedrick EJ, Allen EA, Adali T, et al (2011) Comparison of multi-subject ICA methods for analysis of fMRI data. *Human Brain Mapping* vol. 12 pp. 2075–2095. 10.1002/hbm.21170 PMC3117074 21162045

[80] Kelly RE Jr, Alexopoulos GS, Wang Z, Gunning FM, Murphy CF, et al (2010) Visual inspection of independent components: defining a procedure for artifact removal from fMRI data. J Neurosci Methods. 189: 233–45. 20381530 10.1016/j.jneumeth.2010.03.028 PMC3299198

[81] Lin QH, Liu J, Zheng YR, Liang H, Calhoun VD (2010) Semiblind spatial ICA of fMRI using spatial constraints. Hum Brain Mapp 31: 1076–88. 20017117 10.1002/hbm.20919 PMC2891131

[82] Calhoun VD, Adali T (2012) Multisubject independent component analysis of fMRI: a decade of intrinsic networks, default mode, and neurodiagnostic discovery. *IEEE Rev Biomed* Eng. 5: 60–73. 23231989 10.1109/RBME.2012.2211076 PMC4433055

[83] Yu Q, Sui J, Rachakonda S, He H, Pearlson GD, et al (2011) (2011) Altered small-world brain networks in temporal lobe in patients with schizophrenia performing an auditory oddball task. *Frontiers in Systems Neuroscience* vol. 5 pp. 1–13 21369355 10.3389/fnsys.2011.00007 PMC3037777

[84] Yu Q, Sui J, Liu J, Plis SM, Kiehl KA, et al (2013) Disrupted correlation between low frequency power and connectivity strength of resting state brain networks in schizophrenia. *Schizophrenia Research* vol. 143 pp. 165–171. 23182443 10.1016/j.schres.2012.11.001 PMC3540119

[85] Bassett DS, Wymbs NF, Porter MA, Mucha PJ, Carlson JM, et al (2011) Dynamic reconfiguration of human brain networks during learning. Proc Natl Acad Sci U S A 108: 7641–6. 21502525 10.1073/pnas.1018985108 PMC3088578

[86] Cole MW, Pathak S, Schneider W (2010) Identifying the brain's most globally connected regions. Neuroimage. 49(4): 3132–48. 19909818 10.1016/j.neuroimage.2009.11.001

[87] Teasdale G, Jennett B (1974) Assessment of Command Impaired Consciousness: A Practical Scale. The Lancet. 304: 81–84. 10.1016/s0140-6736(74)91639-0 4136544

[88] Palacios EM, Sala-Llonch R, Junque C, Roig T, Tormos JM, et al (2013) Resting-State Functional Magnetic Resonance Imaging Activity and Connectivity and Cognitive Outcome in Traumatic Brain Injury. JAMA Neurol p 1–7. 10.1001/jamaneurol.2013.38 23689958

[89] Tang L, Ge Y, Sodickson DK, Miles L, Zhou Y, et al (2011) Thalamic resting-state functional networks: disruption in patients with mild traumatic brain injury. Radiology. 260: 831–40. 21775670 10.1148/radiol.11110014 PMC3157002

[90] Di Perri C, Bastianello S, Bartsch AJ, Pistarini C, Maggioni G, et al (2013) Limbic hyperconnectivity in the vegetative state. Neurology. 81(16): 1417–24. 24049132 10.1212/WNL.0b013e3182a43b78

[91] Craig AD (2009) How do you feel—now? The anterior insula and human awareness. N*at. Rev. Neurosci*. 10 (1): 59–70. 19096369 10.1038/nrn2555

[92] Gazzaniga MS, Smylie CS (1984) Dissociation of language and cognition. A psychological profile of two disconnected right hemispheres. *Brain* 107 (Pt 1): 145–53. 6697151 10.1093/brain/107.1.145

[93] Gazzaniga MS, Volpe BT, Smylie CS, Wilson DH, LeDoux JE (1979) Plasticity in speech organization following commissurotomy. *Brain*. 102(4): 805–15. 116711 10.1093/brain/102.4.805

[94] Barabási A-L, Albert R (1999) “Emergence of scaling in random networks”. S*cience* 286 (5439): 509–512 doi:10.1126/science.286.5439.509 10521342 10.1126/science.286.5439.509

[95] Mantini D, Gerits A, Nelissen K, Durand JB, Joly O, et al (2011) Default mode of brain function in monkeys. *J Neurosci.* 31(36): 12954–62. 21900574 10.1523/JNEUROSCI.2318-11.2011 PMC3686636

[96] Liang X, Zou Q, He Y, Yang Y (2013) Coupling of functional connectivity and regional cerebral blood flow reveals a physiological basis for network hubs of the human brain. Proc Natl Acad Sci 110(5): 1929–34. 23319644 10.1073/pnas.1214900110 PMC3562840

[97] Bigler ED (2001) Quantitative magnetic resonance imaging in traumatic brain injury. J Head Trauma Rehabil. 2001 Apr 16(2): 117–34. 10.1097/00001199-200104000-00003 11275574

