# Correction: The Rich Get Richer: Brain Injury Elicits Hyperconnectivity in Core Subnetworks

**DOI:** 10.1371/journal.pone.0113545

**Published:** 2014-11-10

**Authors:** The PLOS ONE Staff

In Table 5, bold formatting was erroneously removed from certain components during the typesetting process. The publisher apologizes for this error. Please see the corrected Table 5 here.

**Table 5:** Most highly connected nodes (hubs) for TBI and HCs at Time 1 and Time 2 (mean degree values)

TBI Time 1 (n = 21)		TBI Time 2 (n = 21)	
Mean Degree	Spatial Component (ID#)	Mean sum of links	Spatial Component
**11.27**	**R ECN (34)**	11.12	Language (46)
10.97	P. Salience (35)	**11.03**	**Sensorimotor (15)**
10.86	Language (41)	10.98	FEF-par (39)
**10.86**	**vDMN (20)**	**10.63**	**PCC/MPFC (25)**
10.55	A. Salience (51)	**10.59**	**R ECN (45)**
**10.53**	**dDMN (25)**	10.57	A. Salience (47)
10.43	dDMN (31)	**10.43**	**R ECN (34)**
10.40	Precuneus (49)	10.24	A. Salience (4)
**10.35**	**Sensorimotor (15)**	10.24	A. Salience (16)
10.27	A. Salience (48)	**10.10**	**vDMN (20)**
10.25	FEF-par (50)	10.06	dDMN (52)
10.12	Auditory (9)		
10.08	PCC/MPFC (30)		
10.06	Auditory (32)		
**10.04**	**R ECN (45)**		
10.03	A. Salience (42)		
9.92	Language (3)		

HC Time 1 (n = 15)		HC Time 2 (n = 15)	
Mean Degree	Spatial Component	Mean sum of links	Spatial Component
10.52	R.ECN (45)	11.06	R ECN (34)
		10.16	Auditory (32)

**Table 5:** The most highly connected nodes determined by cutoff of 9.9 (2 standard deviations above the mean degree for HC data at Time 1). ***Note:*** values for components listed at Time 1, not listed for Time 2 (41 = 9.72; 51 = 8.8; 31 =  9.54; 49 = 8.96; 48 = 9.70; 50 = 8.91; 9 = 9.3; 42 = 9.31; 3 = 9.83) and for Time 2 but not Time 1 (52 = 9.83; 4 = 9.42; 39 = 8.79; 47 =  8.69; 16 = 8.69) were also at least 1 sd above the HC Time 1 mean, but below 2 sd cutoff. Components in **bold** are identical components between time points.
