# Supplementary material for: The Rich Get Richer: Brain Injury Elicits Hyperconnectivity in Core Subnetworks
Source: PLoS One. 2014 Aug 14;9(8):e104021. doi: 10.1371/journal.pone.0104021 (PMC4133194; doi:10.1371/journal.pone.0104021)
Supplement: Table S1 — General graph properties in TBI and HC using an inclusive graph (components: 60). The current table includes “equivocal” components to demonstrate the robustness of the primary findings in Table 3. The TBI sample shows greater, but non-significant increases in global metrics of connectivity (*p<.0.10 during independent samples one-tailed test). Note: no significant results survive corrections for multiple comparisons at alpha = 0.05. (DOC) [file pone.0104021.s001.doc]

**Table S1: General graph properties in TBI and HC using an inclusive graph (components = 60).**

|  | **TBI**  **Time 1**  **Mean**  **(sd)**  **n=21** | **TBI**  **Time 2**  **Mean**  **(sd)**  **n=21** | **TBI Combined**  **Mean**  **(sd)**  **n=21** | **HC**  **Time 1**  **Mean**  **(sd)**  **n=15** | **HC**  **Time 2**  **Mean**  **(sd)**  **n=15** | **HC Combined**  **Mean**  **(sd)**  **n=15** |
| --- | --- | --- | --- | --- | --- | --- |
| **Total Number of Connections** | | 703.47*   |  | | --- | | 260.2 | |  | | | --- | --- | --- | --- | |  | | | 700.71 | | --- | | 276.89 | | | **702.09** | | --- | | **268.57** | | | 606.73* | | --- | | 161.53 | | | 637.93 | | --- | | 227.06 | | | **622.33** | | --- | | **194.3** | |
| **Total Strength of Connections** | | 306.621* | | --- | | 149.18 | | | 308.61 | | --- | | 164.89 | | | **307.61** | | --- | | **157.03** | | | 247.197* | | --- | | 83.60 | | | 270.633 | | --- | | 123.05 | | | **258.91** | | --- | | **103.33** | |
| **Average path length** | | 1.614 | | --- | | 0.19 | | | 1.634 | | --- | | 0.22 | | | **1.624** | | --- | | **0.20** | | | 1.676 | | --- | | 0.13 | | | 1.66 | | --- | | 0.173081 | | | **1.67** | | --- | | **0.155** | |
| Clustering coefficient (weighted) | | 0.238 | | --- | | 0.08 | | | 0.245 | | --- | | 0.08 | | | **0.241** | | --- | | **0.08** | | | 0.212 | | --- | | 0.04 | | | 0.22 | | --- | | 0.07 | | | **0.218** | | --- | | **0.05** | |

**Table S1 Legend:** The current table includes “equivocal” components to demonstrate the robustness of the primary findings in Table 3. The TBI sample shows greater, but non-significant increases in global metrics of connectivity (*p<.0.10 during independent samples one-tailed test). **Note**: no significant results survive corrections for multiple comparisons.
