# Supplementary material for: The Rich Get Richer: Brain Injury Elicits Hyperconnectivity in Core Subnetworks
Source: PLoS One. 2014 Aug 14;9(8):e104021. doi: 10.1371/journal.pone.0104021 (PMC4133194; doi:10.1371/journal.pone.0104021)
Supplement: Table S2 — General graph properties in TBI and HC in Sparse Graph (minimum r-value = 0.403). Metrics for the TBI sample show greater, but non-significant increases in global metrics of connectivity (*p<.0.10; independent samples one-tailed test). Note: no significant results survive corrections for multiple comparisons at alpha = 0.05. (DOC) [file pone.0104021.s002.doc]

**Table S2: General graph properties in TBI and HC in Sparse Graph (minimum r-value=0.403)**

|  | **TBI**  **Time 1**  **Mean**  **(sd)**  **n=21** | **TBI**  **Time 2**  **Mean**  **(sd)**  **n=21** | **TBI Combined**  **Mean**  **(sd)**  **n=21** | **HC**  **Time 1**  **Mean**  **(sd)**  **n=15** | **HC**  **Time 2**  **Mean**  **(sd)**  **n=15** | **HC Combined**  **Mean**  **(sd)**  **n=15** |
| --- | --- | --- | --- | --- | --- | --- |
| **Total Number of Connections** | | 189.52* | | --- | | 122.21 | | | 180.40 | | --- | | 104.12 | | | 184.96 | | --- | | 113.17 | | | 145.6* | | --- | | 74.55 | | | 156.86 | | --- | | 98.33 | | | 151.23 | | --- | | 86.44 | |
| **Total Strength of Connections** | | 131.16* | | --- | | 77.41 | | | 125.33 | | --- | | 66.522 | | | 128.24 | | --- | | 71.97 | | | 102.48* | | --- | | 45.84 | | | 114.11 | | --- | | 59.65 | | | 108.30 | | --- | | 52.74 | |
| **Average path length** | | 1.97 | | --- | | 0.58 | | | 2.17 | | --- | | 0.46 | | | 2.07 | | --- | | 0.52 | | | 2.11 | | --- | | 0.63 | | 1.95  0.48 | | 2.03 | | --- | | 0.56 | |
| **Clustering**  **Coefficient**  **(weighted)** | | 0.439 | | --- | | 0.073 | | | 0.435 | | --- | | 0.074 | | | 0.437 | | --- | | 0.074 | |  | |  | | 0.413  0.051 | | 0.409 | | --- | | 0.058 | |  | |  | | 0.411  0.054 |

**Table S2 Legend:** Metrics for the TBI sample show greater, but non-significant increases in global metrics of connectivity (*p<.0.10; independent samples one-tailed test). **Note**: no significant results survive corrections for multiple comparisons.
